# Circulating Zinc and Copper Levels are Associated with Sperm Quality in Obese Men after Metabolic Surgery: A Pilot Study

**DOI:** 10.3390/nu12113354

**Published:** 2020-10-30

**Authors:** Berniza Calderón, Jesús M. Gómez-Martín, Marta Cuadrado-Ayuso, Pilar Cobeta, Belén Vega-Piñero, Raquel Mateo, Julio Galindo, José I. Botella-Carretero

**Affiliations:** 1 Instituto Tecnológico de Santo Domingo (INTEC), 10602 Santo Domingo, Dominican Republic; dra.bernizacalderon@gmail.com; 2 Affinis, 10131 Santo Domingo, Dominican Republic; 3 Department of Endocrinology and Nutrition, Hospital Universitario Ramón y Cajal, 28034 Madrid, Spain; 4 Instituto Ramón y Cajal de Investigación Sanitaria (IRyCIS), 28034 Madrid, Spain; jmgm82@hotmail.com (J.M.G.-M.); belenvegapi@gmail.com (B.V.-P.); raquel.mateo@salud.madrid.org (R.M.); 5 Department of General and Digestive Surgery, Hospital Universitario Ramón y Cajal, 28034 Madrid, Spain; marta.cuadrado.ayuso@gmail.com (M.C.-A.); pilar.cobeta@salud.madrid.org (P.C.); julio.galindo@salud.madrid.org (J.G.); 6 Department of Anesthesiology, Hospital Universitario Ramón y Cajal, 28034 Madrid, Spain; 7 CIBER de Fisiopatología de la Obesidad y Nutrición (CIBERobn), 28034 Madrid, Spain

**Keywords:** zinc, copper, iron, male fertility, sperm, metabolic surgery

## Abstract

(1) Background: Inadequate levels of several trace elements and vitamins may impair spermatogenesis in men. Although weight loss after metabolic surgery normalizes male reproductive hormones, sperm quality seems to not improve. We hypothesized that circulating concentrations of zinc, copper and other trace elements and vitamins might be involved. (2) Methods: We studied 20 men submitted to metabolic surgery at baseline and after two years. Hormone profiles, serum trace elements and vitamins were studied together with sperm analysis. (3) Results: At follow-up, serum testosterone, follicle-stimulating hormone and inhibin B concentrations increased showing a beneficial hormonal response for spermatogenesis. Conversely, serum copper, zinc and ferritin showed a decline after surgery. In total, 33% of men showed zinc deficiency, 27% copper deficiency and 20% iron deficiency, among others. Sperm analysis showed that all revaluated patients had at least one abnormal parameter. Serum zinc concentrations showed a positive correlation with progressive motility (r = 0.577, *p* = 0.031), and serum ferritin a positive correlation with sperm volume (ρ = 0.535, *p* = 0.049). Serum copper showed a weak and near significant correlation with motility (r = 0.115, *p* = 0.051). (4) Conclusions: The lack of improvement in sperm quality in obese men after metabolic surgery may be related to nutrient malabsorption, especially zinc, copper and iron.

## 1. Introduction

Zinc and copper as well as several vitamins play a role in the development of sperm cells and their protection from oxidative stress, with possible effects on male fertility [1,2]. Besides, obesity is associated with several sperm alterations, such as reduced sperm concentration, altered motility, morphology, DNA fragmentation and abnormal acrosome reaction [3,4,5]. Obesity is also associated with a deficiency of several nutrients, such as zinc, copper, iron, vitamins A, D and E and folic acid. [6]. The causes are probably multi-factorial with low ingestion of vegetables and fruits and high intake of poor nutritional quality foods. Increased adiposity may impair the storage and availability of several nutrients, and the low-grade inflammation state of obesity produces a depletion of antioxidant molecules through an increment in oxidative stress [7].

Metabolic surgery (also known as bariatric or obesity surgery) is a very effective approach for the treatment of excess weight and the resolution of obesity-associated comorbidities [8,9,10], including functional hypogonadism in men [11,12]. However, even though the levels of reproductive hormones are normalized after weight loss [11], the same does not apply to sperm quality and fertility. Previous small studies that included case reports reported no change or even worsening in sperm characteristics after metabolic surgery [13,14,15,16,17,18,19]. A meta-analysis on the effects of metabolic surgery on male reproductive hormones and sperm characteristics showed a beneficial increase in androgens and a decrease in estrogens. On the other hand, the function and quality of sperm did not improve [20], which we have confirmed in a recent study of our group [21].

Therefore, as weight loss after metabolic surgery normalizes androgens and other reproductive hormones but sperm quality seems to not improve, we hypothesize that nutritional derangements that occur after metabolic surgery might be involved. Among these, the circulating concentrations of some trace elements, especially zinc and copper, as well as some vitamins might be associated with this lack of improvement.

## 2. Materials and Methods

### 2.1. Subjects

Twenty men with grade 2 or 3 obesity were included. These patients were part of a larger cohort previously reported [5]. They underwent metabolic surgery (4 patients laparoscopic sleeve gastrectomy and 16 patients laparoscopic gastric bypass) and were followed for two years. Inclusion criteria were a BMI ≥ 35 kg/m^2^ and commitment to attend the follow-up visits. Exclusion criteria were previous diagnosis of thyroid or cardiac disease, kidney or liver failure, hyperprolactinemia, therapies for sexual dysfunction, hypogonadism or any use of antiandrogens and/or GnRH agonist (for prostate disease or alopecia) as well as drugs interfering with gonadal function.

### 2.2. Statement of Ethics

Written informed consent was obtained from every participant. The study was approved by the Institutional Review Board of our Hospital (approval number 333/14—PI 18/00132) and conducted according to the Declaration of Helsinki.

### 2.3. Anthropometric and Analytical Procedures

Between 8 AM and 9 AM and after a 12-h overnight fast, basal blood samples were obtained in each patient. Blood pressure and anthropometric parameters were also recorded and BMI was calculated as weight in kilograms divided by height in meters squared. Waist circumference was measured as the smallest perimeter between the costal border and the anterior suprailiac spines.

Serum glucose was measured by colorimetric methods (Architect ci8200, Abbot Diagnostics, Berkshire, UK). High-density lipoprotein (HDL) cholesterol was measured after plasma precipitation with phosphotungstic acid and Mg^2+^ (Boehringer Mannheim GmbH, Mannheim, Germany). Total cholesterol and triglycerides were measured by enzymatic methods (Menarini Diagnostica, Florence, Italy). Low-density lipoprotein (LDL) cholesterol was calculated by Friedewald’s formula.

Serum ferritin and insulin were measured by immunochemoluminescence (Immulite 2000, Siemens Healthcare Diagnostics Inc., Gwynedd, UK), with a coefficient of variation (CV) <10%. Insulin resistance in the fasting state was estimated by homeostasis model assessment of insulin resistance (HOMA-IR).

Reproductive hormones included luteinizing hormone (LH), follicle-stimulating hormone (FSH), total testosterone (TT) and estradiol. TSH was also measured both before and after surgery by an immune-chemoluminescent method. They were assayed as previously reported [5]. Free testosterone (FT) was calculated from TT and sex hormone-binding globulin (SHBG) using the Vermeulen formula [22]. All assays had CV < 10%. Normal ranges for TT were 250–1000 ng/dL, for SHBG were 117–639 μg/dL and for FT were 6.5–18.3 ng/dL.

### 2.4. Trace Elements and Vitamins Analyses

Serum copper and zinc were analyzed by atomic absorption spectrophotometry (AAnalyst 800, Perkin Elmer, CA, USA). The normal ranges were 75–150 µg/dL for copper and 60–150 µg/dL for zinc. Serum retinol and α-tocopherol were measured by high performance liquid chromatography (HPLC System Gold, Beckman Instruments Inc. Fullerton, CA, USA). Intra- and inter-assay CVs were less than 3%. Reference ranges were 25–80 μg/dL for retinol and 5.7–16.7 μg/mL for α-tocopherol. Serum retinol-binding protein (RBP) was measured by nephelometry (BN-II System, Dade Behring, Siemens Medical Solutions Diagnostics, Munich, Germany). Intra- and inter-assay CVs were <5%. Reference ranges were between 2.5 and 6.5 mg/mL for RBP. Immunochemoluminescence was used for the measurement of folic acid and cobalamin (ADVIA Centaur CP Immunoassay system, Siemens Healthcare Diagnostics Inc., Gwynedd, UK). Intra- and inter-assay CVs were <10%. Normal ranges were 2.8–13.5 ng/mL and 200–732 pg/mL, respectively. A commercial enzyme-linked immunosorbent assay (ELISA) was employed for the measurement of 25-hydroxyvitamin D (IDS Ltd., Boldon, UK). The specificity of this assay is 100% for 25-hydroxyvitamin D3 and 75% for 25-hydroxyvitamin D2. Normal ranges at our center were considered between 20 and 80 ng/mL.

### 2.5. Sperm Analysis

Sperm samples were obtained after a 4-day sexual abstinence period. Sperm concentration was measured by a Bürker hemocytometer. Sperm motility was analyzed by a Sperm Class Analyzer^®^ SCA 2002 (Microptic Inc., Barcelona, Spain). We used the World Health Organization reference values [5,23]: volume ≥ 1.5 mL, concentration ≥ 15 × 10^6^/mL, semen pH ≥ 7.2, total motility ≥ 40% and progressive motility ≥ 32%, sperm viability ≥ 58% and normal morphology ≥ 4%.

### 2.6. Statistics

The included patients in this study are part of a larger cohort previously reported [5]. Therefore, the present sub-study should be regarded as a pilot one, as sample size was calculated in the whole cohort for finding significant changes in testosterone. Post hoc sample size analyses are provided in the Discussion and all calculations were performed with GRANMO 7.12 (Institut Municipal d’Investigació Médica, Barcelona, Spain).

Results are expressed as means ± SD. The Kolmogorov–Smirnov statistic was used to check for normal distributions. An independent Student’s *t*-test or a Mann–Whitney U test was used to compare the central tendencies as appropriate. Comparisons of continuous variables before and after surgery were performed by a paired *t*-test or Wilcoxon test. Bivariate correlations were analyzed by Pearson or Spearman tests as needed.

Analyses were performed using SPSS 18 (SPSS Inc., Chicago, IL, USA). We considered a *p* < 0.05 as statistically significant.

## 3. Results

### 3.1. Baseline Characteristics

The twenty included obese men in this study were 40 ± 8 years old. At baseline, 11 patients (55%) had hypertension, 9 (45%) hyperlipidemia, 6 (30%) type 2 diabetes mellitus and 5 (25%) were smokers. One patient had a previous stroke, whereas none were diagnosed of coronary heart disease. The anthropometric and analytical characteristics of the included patients at baseline and at the end of the follow-up two years after metabolic surgery are shown in Table 1.

### 3.2. Zinc, Copper, Other Trace Elements and Sperm Quality at Baseline

Serum analyses for trace elements showed one man (5%) with low circulating copper and another one (5%) with low circulating zinc, whereas iron deficiency was found in three patients but none showed anemia.

Regarding serum vitamins, eight men (40%) had vitamin D deficiency, three (15%) showed low circulating retinol, one (5%) low circulating alpha tocopherol and two patients (10%) folic acid deficiency. No one had cobalamin deficiency. Serum vitamins concentrations of the obese men at baseline and after metabolic surgery are shown in Table 2.

We found abnormal results in sperm quality in 11 patients (55%), abnormal morphology in 8 (40%)—52% with head alterations and 48% with mixed alterations—abnormal motility in 5 (25%) and low sperm concentration in 4 (20%). Seven (35%) men showed low TT and/or FT and were therefore regarded as having obesity-related secondary hypogonadism (Table 3).

### 3.3. Changes at Follow-Up

After metabolic surgery, all patients were placed on a multivitamin and mineral supplement on a daily basis and blood tests were performed after 3, 6, 12 and 24 months to check for possible trace elements or vitamin deficiencies and treat them appropriately.

At the end of the two years of follow-up, there was a very important and significant weight loss (Table 1). Resolution of obesity comorbidities was achieved in the majority of patients: 9 out of 11 of the hypertensive patients (antihypertensive medication was withdrawn in all of them except for two patients who remained on angiotensin-converting enzyme inhibitors), 5 out of the 6 type 2 diabetic patients (insulin was withdrawn in 3 of them, two others stopped antidiabetic oral agents and one was still on metformin) and 5 out of the 9 patients with hyperlipidemia (4 remained on statins).

A significant difference in TSH levels before and after surgery but with no clinical relevance was found, as all patients had normal TSH values, except for one patient with a very mild subclinical hypothyroidism without thyroid autoimmunity both before and after surgery (TSH levels 5.6 and 5.8 uU/mL, respectively). Serum TT, SHBG, FT, FSH and inhibin B concentrations increased, showing a beneficial hormonal response for spermatogenesis to occur (Table 1).

### 3.4. Zinc, Copper, Other Trace Elements and Sperm Quality after Metabolic Surgery

**Figure 1:**
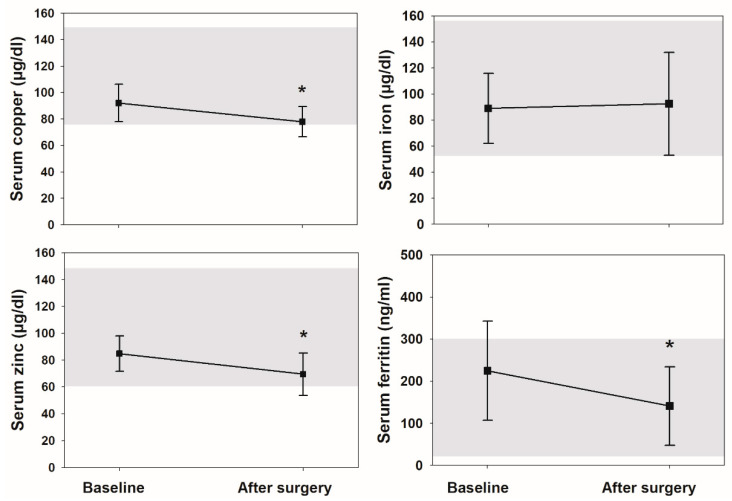
Serum zinc, copper, iron and ferritin, before and after surgery. Data are expressed as means (squares) and SD (error bars). Shaded areas show normal ranges. * *p* < 0.05 from baseline.

Conversely, serum copper, zinc and ferritin showed a decline after surgery (Figure 1). Four men (27%) showed copper deficiency, five (33%) zinc deficiency and three (20%) iron deficiency. Four men (27%) showed vitamin D deficiency, one (7%) retinol deficiency and another one (7%) cobalamin deficiency. No one presented tocopherol or folate deficiencies (Table 2).

At the end of the study, all patients were on a multivitamin and mineral supplement. Eighteen patients were on cobalamin supplements (five of them as a 1-mg monthly injection, the rest of them as 1-mg once-weekly or twice-weekly oral vials). Sixteen patients were on vitamin D (oral capsules providing 0.266 mg of calcitriol once-weekly in nine of them, twice-weekly in four of them and three times per week in the rest). Five patients were on 50,000 units of oral retinol per week (in three patients) or twice-weekly (two patients).

Twelve patients were on daily oral iron supplementation. Three patients needed intravenous iron on a monthly basis with varying doses (between 500- and 1000-mg IV infusions). Oral zinc was needed in six men and oral copper in five, and doses were adjusted in five and four men, respectively, at the last visit of the study (in fact, these men were those previously mentioned with copper and zinc deficiencies).

We then compared the circulating levels of trace elements and vitamins between patients who underwent sleeve gastrectomy (*n* = 4) vs. gastric bypass (*n* = 11). Although the sample size was too small to reach a good statistical power, both retinol and tocopherol were lower after gastric bypass (z = −2.032, *p* = 0.043 for retinol; z = −2.309, *p* = 0.021 for tocopherol). There was also a trend for lower circulating concentrations of 25-hydroxyvitamin D (z = −1.876, *p* = 0.061). On the other hand, we found no differences between trace elements with the type of surgical technique (z = −0.623, *p* = 0.533 for copper; z = −1.663, *p* = 0.096 for zinc; z = −1.156, *p* = 0.248 for iron).

Sperm analysis showed that all revaluated patients had abnormal parameters: 60% had low sperm concentrations, 58% low motility and 63% abnormal morphology. Oligozoospermia was found in nine patients. Sperm volume showed a small decrease and pH a slight increase. The rest of the sperm parameters remained mostly unchanged (Table 3).

**Figure 2:**
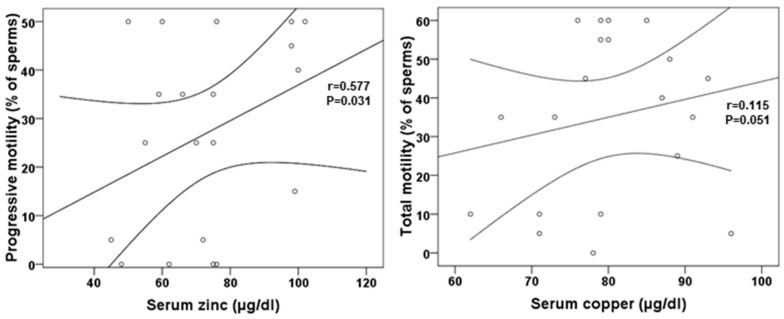
Serum zinc and copper correlations with sperm motility.

Some of the semen parameters showed significant correlations with trace elements. Serum zinc concentrations showed a positive correlation with progressive motility (r = 0.577, *p* = 0.031) and serum copper showed a weak and near-significant correlation with motility (r = 0.115, *p* = 0.051) (Figure 2). Besides, serum ferritin showed a positive correlation with sperm volume (ρ = 0.535, *p* = 0.049), and folate a near significant positive correlation with sperm pH (r = 0.509, *p* = 0.056).

## 4. Discussion

We have shown in the present study that although metabolic surgery normalizes androgens and other reproductive hormones in men, sperm quality does not improve, and this might be related to the malabsorption of several nutrients that occurs in the first two years after surgery. Low circulating concentrations of several trace elements, such as zinc, copper and iron, are frequently observed after metabolic surgery, especially with malabsorptive techniques [24,25].

Zinc, a highly abundant element, is an essential micronutrient. After its ingestion and absorption through the small intestine, it is bound predominantly to albumin as well as to other proteins, including prealbumin, α2-macroglobulin, transferrin, ceruloplasmin, haptoglobin, immunoglobulins and complement. Serum-circulating zinc accounts for 0.1% of total body stores [26]. There are two families of proteins responsible for the transit of zinc across membranes: zinc-importer proteins that transport zinc into the cytosol and zinc transporter (ZnT) proteins moving zinc outside the cytosol [27]. Most of the zinc body content is in bone and muscle tissues, but many other organs also harbor significant concentrations. Interestingly, the prostate, the testes and the epididymis are also rich in zinc [28].

Decreased male fertility has been linked to zinc deficiency [29,30] as its homeostasis is important in human sperm development and function, capacitation and fertilization [26]. Consequently, zinc supplementation improves sexual dysfunction in rats [31] and increases sperm counts in men [32]. However, in a recent multi-center trial with 2370 men, folic acid and zinc supplementation did not improve semen quality or birth rates [33]. Therefore, the actual role of zinc supplementation—dosing, timing and effectiveness—for fertility in men is still a matter of controversy.

Copper and iron are also trace elements that are present in tissues of the male reproductive system acting as essential cofactors [34]. Common dietary sources of copper are meat, shellfish, seeds, potatoes and legumes, whole grains and nuts. After intestinal absorption, it circulates mainly bound to albumin and is transported to the liver. A small amount is excreted in bile and the majority binds to ceruloplasmin—a copper dependent ferroxidase—and is released back into the bloodstream [35]. Within the testes, about 80% of seminal ceruloplasmin is in Sertoli cells and the rest is in metallothioneins (MT) which bind both copper and zinc in Sertoli and also in spermatogenic cells [34]. Several studies in men and also in animals have shown that copper has beneficial effects on semen volume and sperm count and motility [36,37,38]. Copper-deficient animals showed less-developed and -active seminiferous tubules due to Sertoli cell inactivity and they reverted to normal with copper supplementation [39,40]. One of the suggested mechanisms is DNA damage due to upregulated reactive oxygen species (ROS) in these animals [41], as MT and glutathione, both ROS scavengers, are altered in copper deficiency [34].

Iron and copper metabolism are closely related. Ceruloplasmin facilitates the oxidation of ferrous iron into the ferric form and then enables its transport by transferrin. Iron enters the cell by endocytosis via the transferrin membrane receptor, and while in the cytosol, it is taken up by ferritin, the iron storage protein [42]. Low copper levels impair the ferroxidase properties of both ferritin and ceruloplasmin. This in turn may impact sperm quality because Sertoli and Leydig cells are important sources of ferritin, a readily available source of iron for the spermatozoa [34]. Iron in seminal plasma sustains spermatozoa energy metabolism and it is positively correlated with sperm motility [34].

Our results are in agreement with previous knowledge as we have shown a decline in the circulating concentrations of zinc, copper and ferritin (the most important iron storage protein) after metabolic surgery, together with a lack of sperm-quality improvement, despite the normalization of reproductive hormones. It is true that a long period of follow-up is needed to correctly assess the recovery of sperm after an insult, as the average duration of gonadotrophin therapy until the first sperm appears is about 4 months [43]. Besides, sperm induction cycles may take several months before resulting in pregnancy [44,45]. In our study, we revaluated the patients two years after surgery, so we expected some improvement at least. Furthermore, all patients were screened for possible vitamin and trace element deficiencies at follow-up and treated with oral supplements. The fact that many of them had still some deficiencies at the end of the follow-up may be due to intestinal malabsorption. It also implies that, probably, some of them were undertreated, and in fact, zinc and copper doses needed adjustment in some patients at the end of the study.

Although many studies, both in animal models and humans, have shown the relationship of low circulating and/or seminal concentrations of zinc, copper and iron with low sperm counts, evidence supporting the supplementation of these trace elements for treating male infertility is not well-established yet.

### Limitations of the Study

The relatively small sample size is the main limitation of the present study. It precludes from an appropriate multivariate analysis to correct for possible confounding factors on the statistically significant associations that we found for zinc and ferritin with some sperm parameters. Some findings, such as the associations for copper and folate with sperm parameters, did not reach statistical significance, also because of the small sample size. A post hoc sample size analysis shows that at least 25 patients are needed to reach a significant level for the correlation of 0.5 found between folate and sperm pH. In addition, more than 30 patients would be needed to perform a multivariate analysis of the effects of the more relevant independent variables found here (zinc, copper and folate) with sperm parameters.

Although we found some differences in vitamins between the two surgical techniques employed (sleeve gastrectomy and gastric bypass), we did not find differences in copper, zinc or iron, which is, again, a limitation of the sample size of the study. A post hoc sample size analysis shows that at least 9 and 27 patients with sleeve gastrectomy and gastric bypass, respectively, are needed to reach a significant difference in serum zinc levels.

Serum ferritin, but not serum iron, showed a positive correlation with sperm volume. However, ferritin is not only the most important iron storage protein but also an inflammatory marker which has been associated with obesity and the metabolic syndrome [46]. Therefore, as we did not explore all the aspects of iron metabolism, it is possible that the association between ferritin with sperm quality could be confounded by changes that occur after metabolic surgery.

Therefore, this study should be regarded as a pilot one, with preliminary results that need to be confirmed in future larger studies.

## 5. Conclusions

The lack of improvement in sperm quality in obese men after metabolic surgery may be related to nutrient malabsorption, especially zinc, copper and iron. Future research should focus on determining whether routine supplementation of trace elements after metabolic surgery can improve sperm quality.

## Figures and Tables

**Table 1:** Anthropometric and analytical characteristics of the included obese men.

	At Baseline			After Metabolic Surgery			*p*
*Anthropometric characteristics*							
BMI (kg/m^2^)	50	±	10	32	±	7	<0.001
Waist circumference (cm)	144	±	12	106	±	12	0.012
Systolic BP (mmHg)	146	±	15	130	±	19	0.031
Diastolic BP (mmHg)	89	±	16	77	±	13	0.048
*Metabolic parameters*							
Total cholesterol (mg/dL)	193	±	38	170	±	45	0.002
HDL (mg/dL)	39	±	6	49	±	11	0.002
LDL (mg/dL)	146	±	32	106	±	38	0.006
Triglycerides (mg/dL)	154	±	60	92	±	64	0.001
Fasting glucose (mg/dL)	120	±	63	86	±	13	0.010
Fasting insulin (µU/mL)	25	±	13	6	±	2	0.001
HOMA-IR	7.5	±	5.6	1.2	±	0.3	0.001
TSH (µU/mL)	2.3	±	1.3	1.9	±	1.3	0.018
*Reproductive hormones*							
Total testosterone (ng/dL)	364	±	163	660	±	208	0.025
SHBG (µg/dL)	224	±	83	478	±	144	0.001
Free testosterone (ng/dL)	8	±	4	11	±	3	0.005
Estradiol (pg/mL)	37	±	12	36	±	12	0.151
FSH (U/l)	3.5	±	1.1	4.0	±	1.5	<0.001
LH (U/l)	3.5	±	1.0	3.8	±	1.3	0.066
Inhibin B (ng/mL)	0.56	±	0.25	0.70	±	0.24	0.041

Data are means ± SD. BMI: body mass index; BP: blood pressure; HDL: high-density lipoprotein; LDL: low-density lipoprotein; HOMA-IR: homeostasis model assessment of insulin resistance; TSH: thyrotropin; SHBG: sex hormone-binding globulin; FSH: follicle-stimulating hormone; LH: luteinizing hormone.

**Table 2:** Serum vitamins of the obese men before and after metabolic surgery.

	At Baseline			After Metabolic Surgery			*p*
25-hydroxyvitamin D (ng/mL)	24.4	±	10.7	23.7	±	6.8	0.674
Cobalamin (pg/mL)	374	±	106	335	±	119	0.336
Folic acid (ng/mL)	7.5	±	6.2	9.1	±	5.7	0.203
Retinol (µg/dL)	45	±	13	37	±	10	0.675
Retinol/RBP ratio	9.8	±	1.7	9.9	±	1.5	0.232
Alpha tocopherol (µg/mL)	13	±	3	11	±	4	0.090
Alpha tocopherol/total cholesterol ratio	6.7	±	1.3	6.6	±	0.9	0.667

Data are means ± SD. RBP: retinol binding protein.

**Table 3:** Sperm analysis before and after surgery.

	At Baseline			After Metabolic Surgery			*p*
Sperm volume (ml) *	2.5 (1.0)			2.3 (1.8)			0.041
Sperm concentration (10^6^/mL)	18	±	69	12	±	38	0.074
Fluid pH	8.1	±	0.2	8.3	±	0.3	0.046
Motility—total (%)	35	±	35	35	±	50	0.579
Motility—progressive (%)	20	±	30	25	±	35	0.244
Viability (%)	10	±	5	10	±	5	0.550
Normal forms (%)	2	±	4	2	±	4	0.783

Data are means ± SD, except * that is shown as median (interquartile range).

## Data Statement

Restrictions apply to the availability of data generated or analyzed during this study to preserve patient confidentiality or because they were used under license. The corresponding author will, on request, detail the restrictions and any conditions under which access to some data may be provided.
